# Design and Additive Manufacturing of a Passive Ankle–Foot Orthosis Incorporating Material Characterization for Fiber-Reinforced PETG-CF15

**DOI:** 10.3390/ma16093503

**Published:** 2023-05-02

**Authors:** Patrick Steck, David Scherb, Christian Witzgall, Jörg Miehling, Sandro Wartzack

**Affiliations:** Engineering Design, Friedrich-Alexander-Universität Erlangen-Nürnberg, 91058 Erlangen, Germany; steck@mfk.fau.de (P.S.);

**Keywords:** ankle–foot orthoses, additive manufacturing, ankle splints, fused layer modelling, topology optimization, fiber reinforcement, material characterization, user-centered design

## Abstract

The individualization of patient-specific ankle joint orthoses is becoming increasingly important and can be ideally realized by means of additive manufacturing. However, currently, there are no functional additively manufactured fiber-reinforced products that are used in the field of orthopedic treatment. In this paper, an approach as to how additively manufactured orthopedic products can be designed and produced quickly and flexibly in the future is presented. This is demonstrated using the example of a solid ankle–foot orthosis. For this purpose, test results on PETG-CF15, which were determined in a previous work, were integrated into a material map for an FEA simulation. Therewith, the question can be answered as to whether production parameters that were determined at the test specimen level can also be adapted to real, usable components. Furthermore, gait recordings were used as loading conditions to obtain exact results for the final product. In order to perfectly adapt the design of the splint to the user, a 3D scan of a foot was performed to obtain a perfect design space for topology optimization. This resulted in a patient-specific and stiffness-optimized product. Subsequently, it was demonstrated that the orthosis could be manufactured using fused layer modelling. Finally, a comparison between the conventional design and the consideration of AM-specific properties was made. On this basis, it can be stated that the wearing comfort of the patient-specific design is very good, but the tightening of the splint still needs to be improved.

## 1. Introduction

### 1.1. Motivation

Due to the progressive aging of our society, more and more people are dependent on medical aids such as orthoses, protheses, and splints, which has led to a growing market for medical devices. Orthoses are divided into different categories (active: with an external energy supply; passive: without an additional external energy supply), which, in turn, are suitable for different applications (splinting, mobilization, etc.) [1]. They are used in the field of remobilization, especially physiotherapy and the general treatment of pathophysiological diseases. The present work is focused on the fabrication of a so-called solid-ankle–foot orthosis (SAFO), which is often referred to as a leg splint, as is the case in this paper [2]. These are used in the treatment of ankle fractures. However, most medical aids do not fit a patient’s limb perfectly due to the fact that they are mostly mass-produced [3]. In the case of devices that are intended to be used for a long or even lifelong period, the wrong ergonomics leads to relieving postures, secondary diseases, and, in the worst case, to incorrect healing of bone fractures [4]. This was also proven by a study by Abzug et al. [5] in which 59% of the study participants suffered from secondary diseases due to improper fitting or non-fitting of leg splints. The main reason why ill-fitting aids cause long-term problems for a user is the constantly changing load case due to displacements and the associated poor/incorrect load application on bones and joints. One possibility to automatically generate medical aids adapted to the patient is the integration of topology optimization methods into the product development process [6]. Together with a previous load determination by means of motion recordings with parallel pressure plate measurements, the load collectives can be determined precisely [7]. Through the use of the additive manufacturing (AM) technology fused layer modelling (FLM), which allow for a great deal of design freedom, the organic and individual design proposals obtained from topology optimization can be easily manufactured. When it comes to a need for fiber-reinforcement (FR), FLM is an especially good choice, because it considers the specific load trajectories inside a structure through aligning the print paths in the same direction as the load vector [8]. However, there are no usable additively manufactured and topology-optimized FR products in the field of medical treatment.

### 1.2. State of the Art

AM processes are characterized by their high degree of design freedom [9,10]. Furthermore, the production price is independent of the number of pieces to be produced, which simplifies the cost calculation for design adaptions and product replacements [11]. In order to be able to start production quickly and universally, flexible and cost-effective manufacturing processes should be used. In this case, stereolithography (SLA) and fused layer modelling (FLM) are the most widespread variants in AM [12,13]. However, due to the liquid form of the raw material, there is no possibility of aligning reinforcing fibers such as carbon or glass fiber. FLM does not have these disadvantages, but due to the melting process characteristics, it is limited exclusively to thermoplastics [11]. Compared to duroplastics, these have a lower melting and softening temperature. Nevertheless, through even more advanced research in the field of additively usable polymers, it is now possible to use high-temperature thermoplastics such as polyetheretherketones (PEEK) and polyamides (PA). This is why the FLM process is becoming more and more frequently used for industrial applications [14,15,16]. Embedded carbon fibers in these materials can help to further improve the stiffness and strength properties [17,18]. The low stiffness losses in topology optimization methods can, thus, be compensated. Furthermore, the work of Prüß et al. [19] shows that the use of AM with FR plastics has great potential for functional integration, especially in the field of medicine and sports medicine. Boolos et al. [20] show a way to manufacture orthotic leg braces with 3D printing. However, the authors present a method in which fixtures are flexible for different body shapes but do not fit perfectly to a patient’s body shape. The work of Borstell et al. [21] introduces a similar 3D scanning approach for a methodical left-hand design to support finger movement while playing a contrabass. The method is based on a common product development process according to Pahl/Beitz [22]. Jin et al. [23] conducted an overall review of additive manufacturing opportunities in orthoses and protheses. The study concludes that there are still clinical, financial, and technological barriers to the full-scale implementation of AM in a service system for custom orthoses and prostheses. Further research in the sectors of AM and topology optimization of orthoses was conducted, for example, by Leary et al. [24], Lin et al. [25], Lin et al. [26], and van Lieshout et al. [27]. In previous work, material testing and characterization were performed by Witzgall et al. [28,29] to provide the basis for usable AM and topology-optimized FRP products. In addition, the study of Valvez et al. [30] shows that when fibers were added to plastics, higher stress relaxations and compressive displacements were observed. Further studies that examined the abrasive behavior and the maximum bending strength of reinforced polyethylene terephthalate glycol (PETG) were published by Hriţuc et al. [31] and Lancea et al. [32]. Finally, Steck et al. [33] described a methodic approach for designing passive lightweight orthoses incorporating human–machine interactions.

### 1.3. Novelty of This Contribution

The novelty of this contribution is, firstly, the combined use of a heuristic topology optimization method with an anisotropic material model and the AM technology FLM on a usable leg splint. Secondly, the manufacturing process and the behavior of the used material, i.e., PETG-CF15, is closely monitored and documented. Therefore, the question that should be answered in this contribution is as follows: can additive manufacturing parameters determined for the manufacturing of test specimens also be used/adapted to the manufacturing process of usable components? The material data determined in a previous paper were, therefore, used as input parameters in a static structural simulation. The manufacturing parameters from the production process of the test specimens were also used as input parameters for the production of the leg splint.

## 2. Materials and Methods

**Figure 1:**
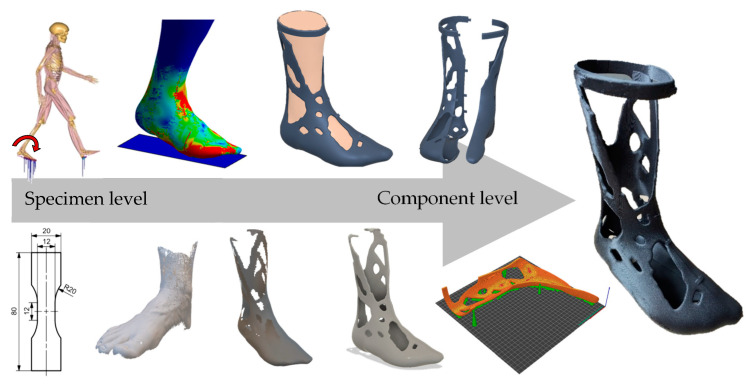
Methodic approach to the development of personalized AM leg splints.

The general procedure for the development of the AM leg splint essentially follows the design for additive manufacturing (DfAM) framework according to Tang et al. [34]. The process is schematically shown in Figure 1 below.

First, the previously determined material data were implemented in the ANSYS simulation software (2022 R2, Ansys Inc., Canonsburg, PA, USA). Furthermore, the loads and constraints were determined on the basis of motion recordings of the test persons. Then, the design spaces and avoidance spaces were defined and the loads and constraints were described. During the design stage, a heuristic topology optimization method developed at the institute was used to optimize the design spaces by iteratively removing mass [35]. The remaining mass described a maximum stiff state with respect to the defined load spectrum. The resulting structure was then implemented again in ANSYS and verified against the load spectrum.

In the design stage, a continuous and AM-compliant model was created from the unusable generic design proposal. Here, a feedback method developed by Mayer et al. [36] was used. Finally, in the elaboration procedure, the now AM-compliant part was loaded into the slicing software PrusaSlicer (2.5.0, Prusa Research, Prague, Czech Republic). There, manufacturing parameters were assigned to the model and build space orientation was defined. The output was a processing routine (G-code) that could be interpreted by the 3D printer used (Raise 3D Pro 2 Plus). The methods that were used are described in more detail below.

### 2.1. Determination of Orthotropic Material Data for PETG CF15

**Figure 2:**
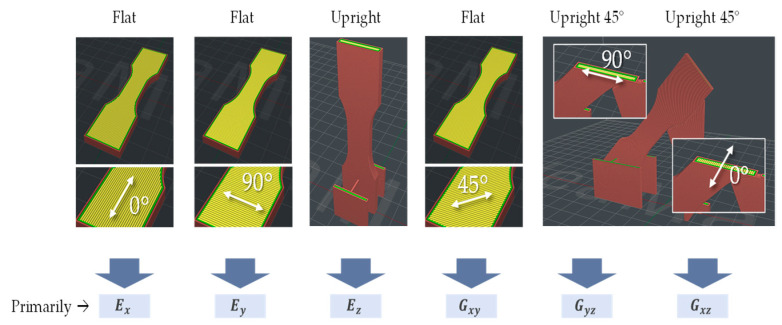
Specimen orientation used for characterization experiments.

The material behavior of additively manufactured, fiber-reinforced plastics is characterized by significant anisotropy. On the one hand, this is due to the fiber reinforcement itself; on the other hand, additive manufacturing with the FLM process already causes the properties of unreinforced plastics to depend on the building direction. Thus, firstly, characterization techniques were used that had previously also been used for injection-molded plastics [37,38,39] or fiber-reinforced laminates [40,41]. In addition, the particularly pronounced material weakening in the height direction of the FLM build space was investigated. Therefore, specimens were required that had been manufactured perpendicularly in the direction of the build height; see Figure 2.

Thirdly, specimens were manufactured diagonally standing in space with different internal orientations. All specimens were filled with solid material, i.e., no diamond or gyroid fill patterns were used.

**Figure 3:**
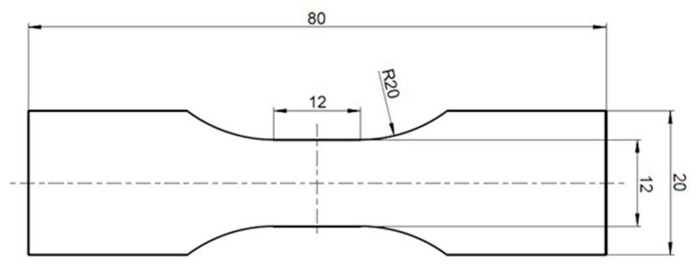
Dimension of the used specimens.

All tests were carried out using tension rods according to Figure 3. The material model was calibrated with the aid of the ANSYS software (2022 R2, Ansys Inc., Canonsburg, PA, USA).

The stiffness parameters evaluated directly from the tensile tests served as the initial values for subsequent model calibration by means of simulation models. Finally, the identified material model was validated by bending tests on a ribbed beam. The material parameters determined in this way, which were also used in the anisotropic topology optimization method presented here, are listed in Table 1. In this table, $E_{x,y,z}$ stands for the Young’s moduli, $G_{xy,yz,xz}$ for the shear moduli, and $\nu_{xy,yz,xz}$ for the corresponding Poisson ratios. The characterization procedure and the corresponding simulation approaches can be found in a previous paper by Witzgall et al. [28].

### 2.2. Specification and Collecting of Patient-Specific Data

**Figure 4:**
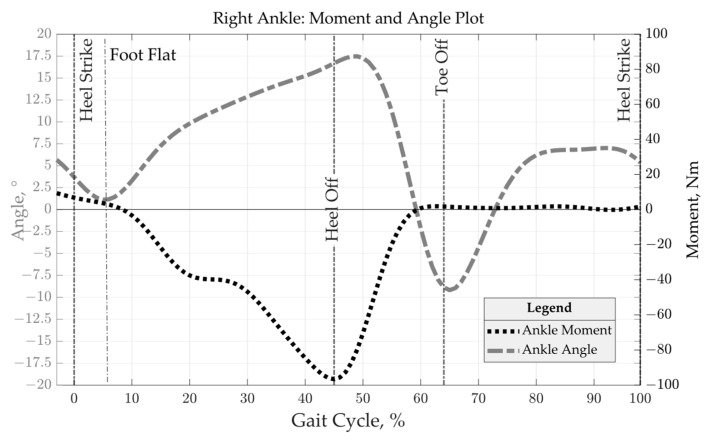
Foot ankle angle and moment distribution over gait cycle.

During the planning phase, input data such as various user-specific parameters, were determined. These were used to define the loads and constraints within the analysis and structural optimization process. In the work of Scherb et al. [7], data concerning the author’s (Patrick Steck) gait behavior were collected for this purpose, which are now being used to develop a stiff leg splint that is suitable for the load. Figure 4 shows the joint moment and joint angle of the right ankle during one gait cycle.

Within the gait cycle, the maximum load distribution is located between the foot flat moment and the heel off moment. Therefore, a static analysis was performed during the heel touches the ground in order to obtain load trajectories and principal stress vectors for the subsequent anisotropic topology optimization process. This is explained in more detail in Section 3.1. 

**Figure 5:**
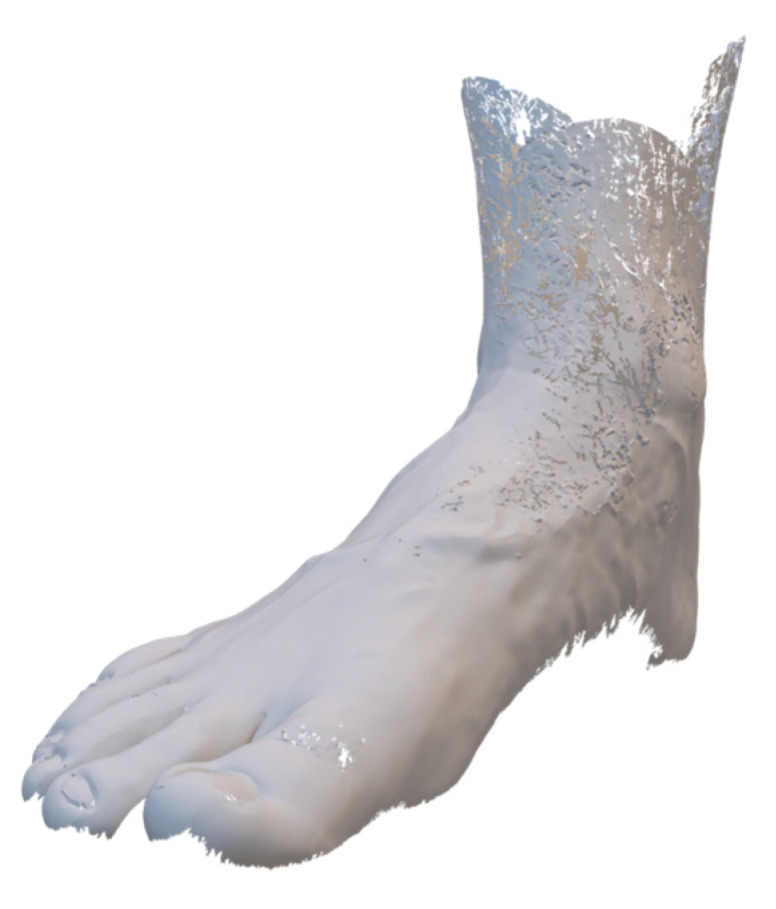
Strip-light projection scanned right foot of the author.

Another important input parameter for topology optimization is the design space. Since a foot is a complex free-form geometry, which is also individually different, a virtual, tessellated image of the foot was created with the aid of a 3D scanner. The scanning method used was an ATOS Compact Scan 12M camera system from Zeiss GOM Technology (Carl Zeiss AG, Oberkochen, Germany), which works with the fringe light projection method. The advantage of this method is the high resolution [42]. However, moving structures are difficult to measure, which is why the foot must be held very still during the measurement. Figure 5 shows the scanned foot of the author.

As can be seen, even veins and the position of the toes can be precisely identified. It is also possible to determine the position of the ankle joint axis through the ankles. In addition, further images of the underside and calf were created and merged using image correlation to generate a complete image of the foot including the leg. The next chapter describes how a static structural analysis was performed with the input data.

### 2.3. Transformation of the Unregulated Mesh into a Simplified and Homogeneous Model

**Figure 6:**
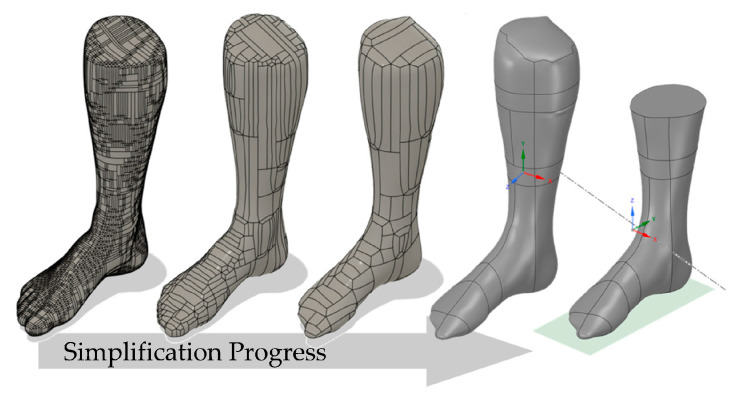
Progress of simplification of the right foot of the author.

After the scanning process, the tessellated foot model was converted into a continuous boundary representation (B-Rep) shell model in order for the model to be able to read in ANSYS as a build space. Simplifications were made in the process. The gaps between the toes were filled and the surface was smoothed. This was done using a script-based feedback method according to Mayer et al. [36] (see Figure 6).

The feedback method is based on a medial-axis method, in which the center surfaces are searched for by continuously shifting the outer surfaces inward. These are then transformed into B-splines and a continuous structure is built up along them. This was performed in several iterations with varying degrees of fineness until a good compromise between the level of detail and simplification was achieved. Finally, the individual surfaces were manually merged and further instantiated in the SpaceClaim program (2022 R2, Ansys Inc., Canonsburg, PA, USA). The coordinate system, ankle axis, and ground contact were then properly oriented and determined. Finally, the design space was shortened in order to allow for the build space of the 3D printer (Raise 3D, Irvine, CA, USA) used. Next, the simplified foot design space was integrated into Ansys and the material data of the PETG-CF15 material was implemented into a material card of Ansys.

### 2.4. Meshing of the Simplified Foot Model

**Figure 7:**
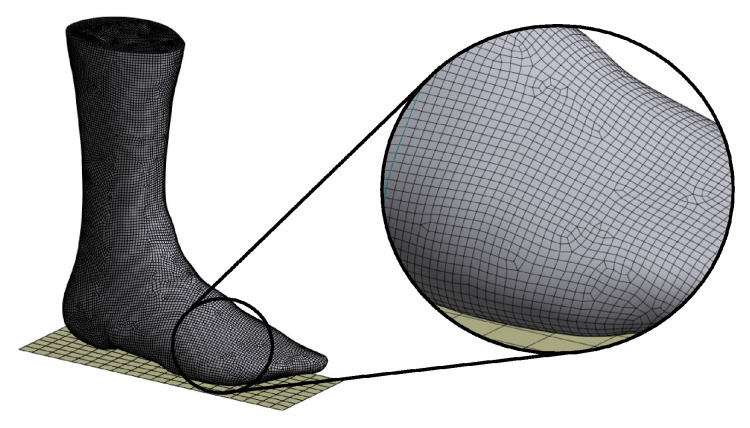
Meshing result for the simplified foot model.

After the foot model was simplified, it was loaded into ANSYS and meshing was performed. A quad/triangle method was used, which resulted in an element size of 2.5 mm for the entire mesh. The resulting mesh can be seen in Figure 7.

Meshing resulted in an almost uniform mesh with a few isolated triangles. Manual contact meshing led to a reduction in element quality, which is why it is not recommended. The mesh had 19,875 nodes and 19,907 elements with the element type SHELL181.

### 2.5. Definition of Input Parameter for Static Structural Analysis

**Figure 8:**
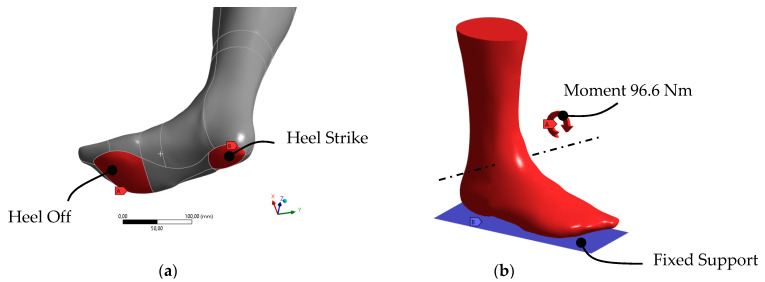
Analysis pre-processing: (**a**) contact region definition; (**b**) loading conditions.

After meshing, the loads were applied and the contact areas defined. Figure 8 shows the defined load spectrum.

**Figure 9:**
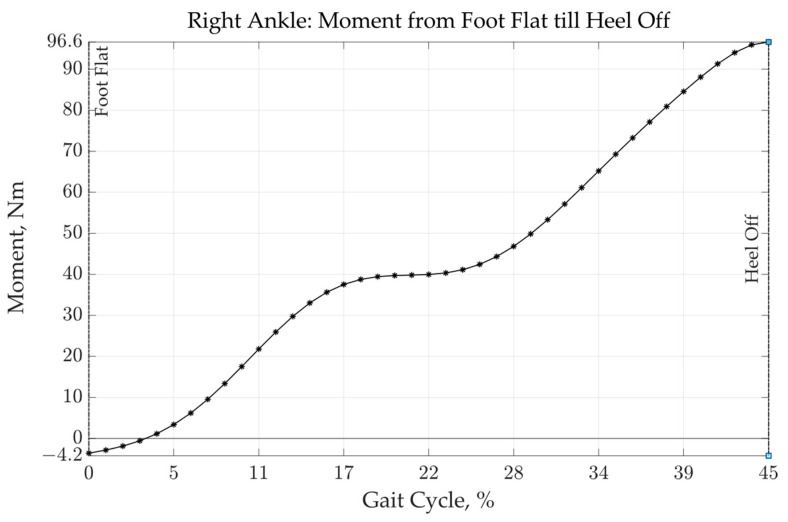
Isolated moment of gait from foot flat until heal off.

Figure 8a shows the contact areas with the base plate. Although the contact points for heel strike and heel off only occurred at the beginning of the foot flat moment, only the heel off was decisive for the analysis of the maximum loads. After heel strike, the ball of the foot moved towards the ground plate. At contact (foot flat), the foot rolled off and lifted the heel up (heel off) as a result of the passing of the body’s center of gravity (midstance). From this point on, the ankle moment around the ankle axis (see Figure 8b) gradually increased until the toes were lifted (toe off). Due to the forward acceleration of the body from pushing off of the ground plate, a maximum torque was observed around the ankle axis. This heel-off torque curve, which is shown in Figure 4, is now shortened as an isolated input variable in Figure 9.

Since a transient structure analysis could not provide valuable insights into the stress distribution within the structure and there are no efficient topology optimization algorithms that can take the transient results into account, optimization was only performed for the maximum moment of 96.6 Nm. 

### 2.6. Heuristic Topology Optimization of a Solid Leg Splint

**Figure 10:**
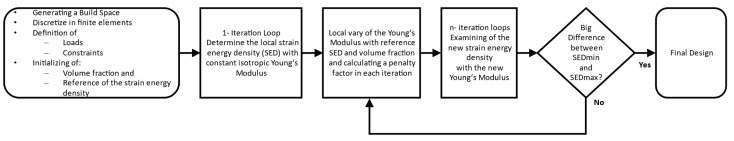
Principal overview of the topology optimization process according to Völkl et al. [35].

The SIMP (solid isotropic material with penalization) [43,44] variant developed by Völkl et al. [35] uses a parallel CAIO (computer-aided internal optimization) [45,46] method to extend the results from the topology optimization process in each iteration so that the element coordinate systems are aligned in the direction of the largest principal stress vector. Basically, the optimization method follows the process in Figure 10.

The strain energy was used as the objective function. If the sum of the strain energies of all elements was minimized, the result was a maximum stiff structure. As constraints, the mentioned fiber orientation and the volume were defined. The target volume was defined as 50% to obtain a solution that weighed half as much as a full volume orthosis.

### 2.7. Process of Fused Layer Modelling of a Solid Leg Splint

**Figure 11:**
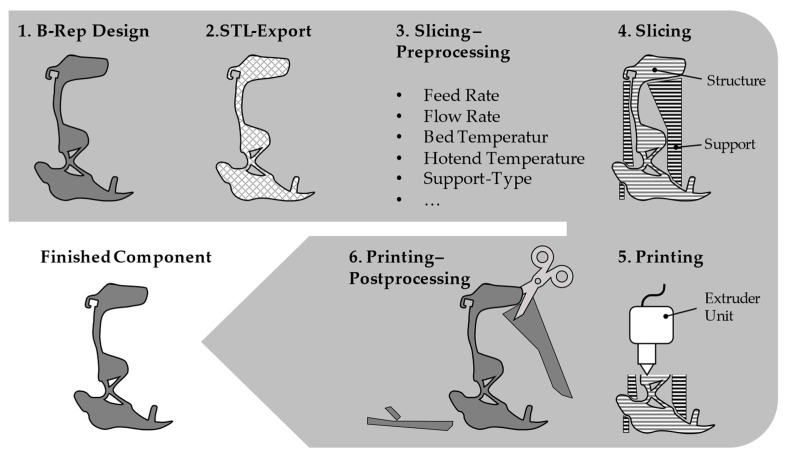
Procedure for the production of leg splints using FLM.

The FLM process is a way to additively manufacture structures from thermoplastics. With the help of a stepper motor, the plastic is extruded specifically and continuously into the melting zone until the desired product is created. Each FLM process basically follows the procedure shown in Figure 11.

In the first step, the modelled component was exported into a tessellated format, in the best case, within the CAD program (Fusion360 2.0.13162, Autodesk, San Francisco, CA, USA). Although the format STL was usually used here, the 3mf format, which was specially developed for 3D printing, has been gaining acceptance for some years. The file was then transferred to a slicing program (PrusaSlicer in this work, 2.5.0, Prusa Research, Prague, Czech Republic). 

## 3. Results

In this chapter, the application of the described methods and the obtained results are explained in more detail. The scheme shown in Figure 1 was followed and the static structure analyses were carried out first. Subsequently, a heuristic topology optimization method was applied and the results are described. The generic design proposal was then manually reworked to ensure the functionality of the product. Finally, the leg splint was additively manufactured. The obtained findings during the monitoring of the manufacturing process and during the examination of the finished product are described by means of a product screening protocol.

### 3.1. Static Structural Analysis on The Foot Demonstrator

**Figure 12:**
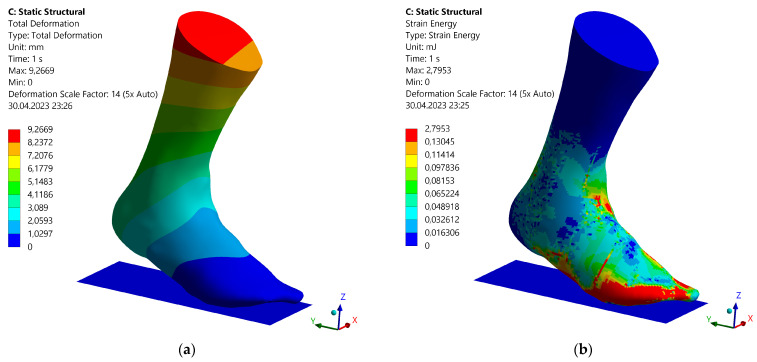
Results from the static structural analysis of the right foot model: (**a**) total deformation; (**b**) strain energy.

When all the preliminary work was done, the static structural analysis was started. Figure 12 shows the results of the analysis. The units used in Figure 12a are mm, and in Figure 12b, they are mJ.

As expected, the foot model deformed around the ankle joint axis. The greatest deformation occurred at the highest point of the calf. This is in contrast to the strain energy distribution, in which the greatest strain energy occurred at the ball of the foot or at the contact surface to the toes. Furthermore, an increase in strain energy was found on the dorsum of the foot at the transition to the lower leg. Medium strain energies were distributed on the dorsum of the foot and the inner and outer instep. Almost no stretching energies occurred on the calf. 

### 3.2. Topology Optimization as a Helpful Tool for Medical Devices

**Figure 13:**
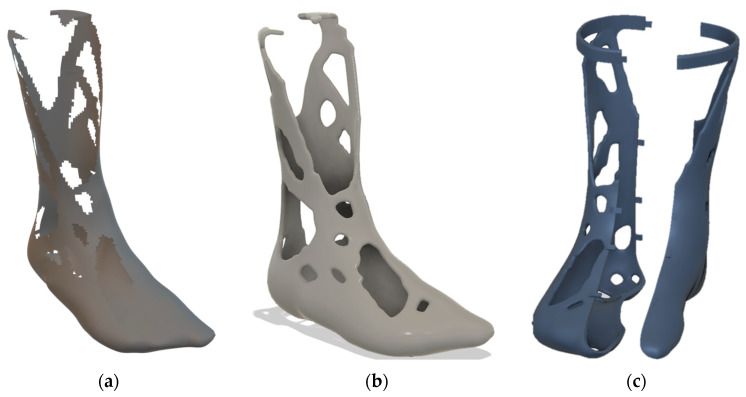
Topology optimization result: (**a**) shell result; (**b**) B-Rep model 5 mm thick; (**c**) wearable split model.

Following the structural analysis, a heuristic topology optimization method according to the method described in Section 2.6 was started with the material data from Section 2.1. Figure 13 shows the topology optimization result, the feedback, and the finished leg splint.

Figure 13a shows the result from the optimization process. The result was converged after 11 iterations. The 60° truss struts are very noticeable, which formed on the inner and outer side of the calf. These are typical for components subjected to tension–compression (bending). In addition, this orientation fits quite well with the DfAM recommendations for a good AM design. Similar results can be found, for example, in Bendsøe et al. [47]. Since the design proposal was a shell solution and discrete holes were created due to the STL export, the result had to be thickened and transferred into a B-Rep model. For this purpose, the method according to Mayer et al. [36] was used once again. Figure 13b shows the reconstructed model thickened to 5 mm. This model could now be loaded into a CAD program, such as Fusion360, and prepared for fabrication. To make the leg splint wearable, it needed to be split. Snap hooks, screw connections, or hinges help to lock the leg splint and fix both halves during use. In this example, snap hooks were constructed to ensure quick donning and doffing of the greave. Furthermore, a belt buckle was added to the top of the leg splint for a better fit at the calf. Figure 13c shows the finished leg splint design. Because the topology optimization process allowed the material to grow only where stress occurred, the resulting structure was more efficient in material usage than solid structures. Another positive side effect of the reduced material was the improved wearing comfort, which resulted from the lower weight.

### 3.3. Slicing Process of the Topology-Optimized Solid Leg Splint

After that, several manufacturing parameters and slicing methods were set in preprocessing. Table 2 shows the manufacturing parameters that were identified in advance for the FormFutura CarbonFil PETG-CF15 [48] material used (matrix material PETG with 15 wt.% short carbon fiber) in the previous work.

**Figure 14:**
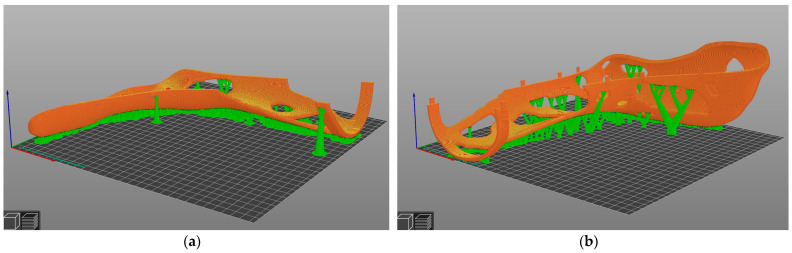
Sliced leg splint in PrusaSlicer (green organic support structure; orange outer skin of the leg splint): (**a**) outer shell; (**b**) inner shell.

After setting the above parameters in PrusaSlicer and starting the slicing sequence, the following slicing preview was obtained (see Figure 14):

The green structures are organic support structures designed to support overhangs with an angle >45°. The building orientation was chosen lying down to better orient the carbon fibers in the loading direction. The Arachne algorithm varied the extrusion quantity for non-uniform wall thicknesses and pressure widths that are not multiples of the set path width, so that a homogeneous pressure structure was created in the pressure plane. This is particularly advantageous for filigree areas such as the snap-fit hooks. Furthermore, a layer height of 0.2 mm was selected, as this was expected to provide a good compromise between total printing time and surface finish. The printing temperatures for the bed and hotend were taken from the data sheet [49]. The speeds were the same as those in the previous work [28]. The infill density was selected as 100%, since, on the one hand, the material had already been saved in the topology optimization process and, on the other hand, the leg splint needed to be designed to be as stiff as possible. With the concentric infill pattern, the perimeters were projected further and further inwards with an offset (=path width). During extrusion, the fibers were oriented along the extrusion direction, which, together with the concentric infill, led to a stiffening of the product. 

After the slicing process, the finished G-code was loaded onto the FLM 3D printer. The printer used in this work was a Raise 3D Pro 2 Plus. This has a build platform of 305 × 305 mm and a maximum build height of 600 mm. The printer is equipped with a dual printhead system, which makes it possible to start with the second head and a new filament roll after one filament roll has been used up. Thus, material-intensive work can be realized. The investigation of the manufacturing process and the results from a visual assessment and practical use are described in more detail in the next chapter.

### 3.4. Additive Manufacturing of a Stiff Leg Splint with Fused Layer Modelling

**Figure 15:**
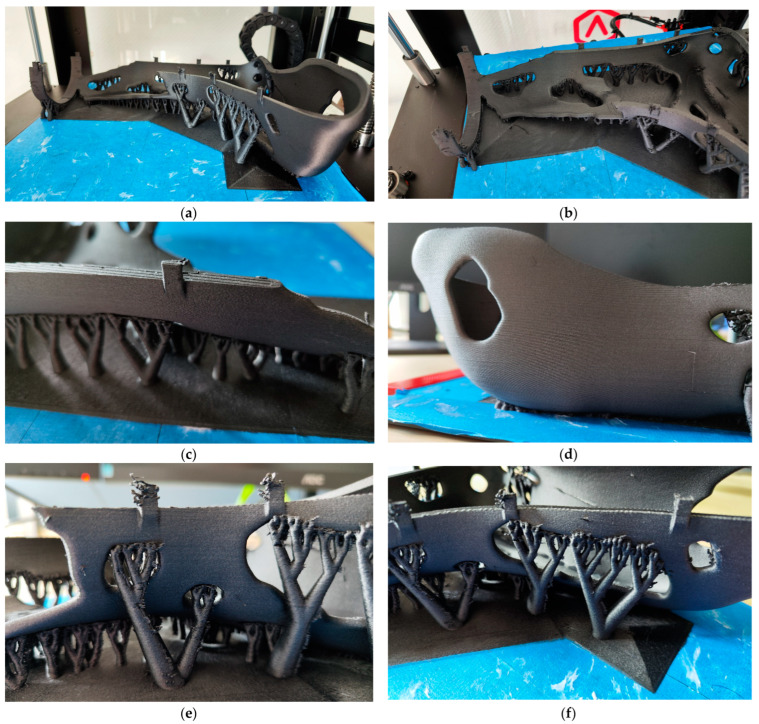
Fabrication of the greave using the outer shell as an example: (**a**) front view; (**b**) top view; (**c**) snap hook; (**d**) bottom view; (**e**) support structure; (**f**) isometric view.

One of the core objectives of this paper was the screening or monitoring of the manufacturing process to find out whether manufacturing parameters for specimens were adaptable to real components. This is now examined in more detail in this chapter. Figure 15 shows the finished printed outer switch of the leg splint on the printing plate.

After the start of the printing process, the first layer was printed. This is critical, especially with PETG-CF15. If it does not adhere perfectly to the printing surface, the product comes off and the print is lost. To ensure good adhesion throughout the entire printing process, a so-called BlueTape from the company 3M was used. Its structure increases the surface area and its blue color provides a good color contrast to the dark material. Furthermore, a conventional glue stick was used as an adhesion promoter to ensure greater adhesion between the product and the build plate. This preparation was necessary because, unlike with the test specimens, support material had to be used to realize overhangs, and these have a small contact area.

For the first layer, the extruder unit moved at 8 mm/s at a flow rate of 100% over the printing plate and extruded the PETG-CF15 material evenly onto the printing surface. This required 1 h 32 min 14 s. Since the small contact area of the support structures caused the print to break off during the first print (the support structures fell off the plate), a specially designed brim was developed in the second iteration to create a connection in the first layer between the individual tree structures (see Figure 15b,e). After this adjustment, the printing of the first layer went smoothly. From the second layer on, the speed could be increased to 25 mm/s and the flow rate could be reduced to 92%. The flow rate had to be reduced, since it was not possible to print the first layer at the same speed. The flow rate had to be reduced because, due to air pockets between PETG and the short fibers, the effective extrusion volume increased after the material was deposited on the printing surface. Further production initially went very well. After one roll of filament (250 g) was used up, printing was interrupted and a new roll had to be loaded into the printer. However, due to customized slicer settings in PrusaSlicer, the print was set to the incremental (relative) extrusion quantity calculation by default, and after a print stop, the Raise 3D Pro2 Plus switched the extrusion to absolute via the firmware, so no further filament was extruded. However, the print could be saved by first determining the effective height at which the extruder stopped extruding and then manipulating the G-code. In this, a comment with information on the current layer height was inserted at every layer change. This comment was searched for and all movements from homing up to this comment were deleted. Then, the M82 command was inserted once again before the shift information and the code was reloaded into the printer. The printer then continued to process the job until the end without any problems. 

After production, the finished leg splint was removed from the printer together with the build plate. The product was removed from the build platform with the aid of a spatula. The tree structures and the brim were then removed from the product. A visual inspection did not reveal any defects. The surface of the rail was very even and without visible blowholes. The fibers in the material provided diffuse light refraction, which concealed minor imperfections and made the surface appear matte and likely injection-molded. The mechanical properties were not tested. However, a simple manual test simulating the load case showed high stiffness. Since, as noted, no significant flaws or structural changes were observed, adequate strength was predicted. After the assessment, the leg splint was ready for use. 

**Figure 16:**
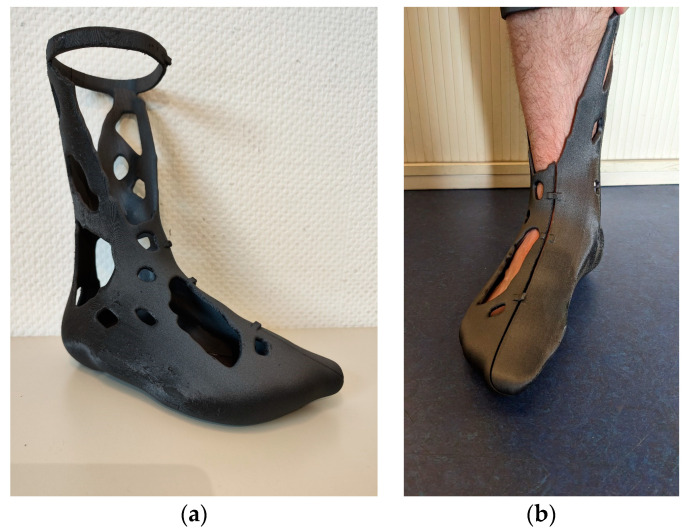
Finished topology-optimized PETG-CF15 leg splint: (**a**) perspective view; (**b**) tightened on the right foot.

Tightening of the leg splint proceeded without incident. Snapping of the snap hooks was possible in principle, but no major deformation was possible due to the increased stiffness caused by the fibers. As a consequence, some snap hooks broke off when trying to push them over the locking mechanism. However, the remaining snap hooks were able to hold the leg splint in place—the wearing comfort of the splint on the bare foot took some getting used to. However, due to the selected build space orientation, all support structures were on the outside of the leg splint, which meant that the inside was very smooth. Figure 16 shows the finished leg splint.

## 4. Discussion

The results are positive for the desired goals of the paper. In addition, during the investigation, there were some interesting findings, which should be considered in future research in the field of additive manufacturing of fiber-reinforced composites.

For example, in the design of the leg splint, manufacturability and usability should not be considered in isolation, but rather should be understood as interacting requirements. “Fuzzy skins”, “ironing”, and other surface functions in slicers, for example, can help produce additively manufactured and functionally integrated hook-and-loop fasteners. Pre-processing functions, which are intended to ensure shape fidelity or higher adhesion, can be integrated directly into the design process and, thus, take on a further user-centered function. Another advantage resulting from this would be a more resource-efficient production, since the support material, for example, is not thrown away but can remain as part of the product. Another design aspect that should be investigated further is the snap-hook connection. Since the stiffer material of the hooks does not allow large deformations, but, at the same time, this material is necessary for the maximum stiffness of the leg splints, a trade-off between the stiffness and necessary deflection should be sought. Stiffness is dependent on the geometry of the structure in addition to Young’s modulus, so lengthening and thinning the snap-fit will likely increase deflection. However, thin structures consist of fewer layers of material. Since interlayer adhesion has a significant effect on the strength of the material, it should be considered in further research. Furthermore, the connection to the calf should be improved in the design. The surface area was increased by adding a strap. However, the connection to the rest of the topology-optimized structure should be increased by means of radii in order to further ensure the support in the event of unpredictable load cases (for example, lateral movement) in the gait.

Due to irregularities in the flatness of the build platform, the first layer also had uneven path widths. Since the extruder unit ran in-plane with an accuracy of 0.01 mm, this meant that the path width varied for the extruded material. However, since the Arachne algorithm additionally led to a variable extrusion amount, the final shape of the first layer was ultimately arbitrary. To be able to counteract this in the future, shape-accurate building platforms with low coefficients of thermal expansion must be used. With regard to the pressure drop, further work should take care to avoid using incremental extrusion M83 when slicing the structure. Since many proprietary slicers have the option of defining a user-defined G-code, it is generally recommended to enter the command for absolute extrusion M82 by default. Since the further printing process went very well, this shows that the previously predicted adaptability of manufacturing parameters from the test specimens to the finished products can be assumed.

Since the leg splint was also used after production, the following can be recommended about wearing comfort: The layer height of 0.2 mm was not noticeable. In the future, thicker layer heights could even be used here, which could lead to shorter production times, especially with the very low printing speed. Nevertheless, an offset to the foot should be planned for further work in order to explore the possibility of inserting a softer and warming (insulating) layer (possibly textile material) between the body part and the splint. The holes resulting from topology optimization had a positive side effect on the ventilation of the splint and are, therefore, particularly suitable for higher ambient temperatures.

## 5. Conclusions

Finally, this paper demonstrates that medical products—and in particular leg splints—can be manufactured using additive manufacturing. Design proposals from topology optimization tools, in particular, are often very organic in their appearance and have undercuts, which makes it difficult or even impossible to manufacture them using other manufacturing processes. Since body parts have complex shapes and acute help is needed in war or disaster areas, for example, the inexpensive and flexible manufacturing approach of fused layer modelling can help to produce fast splints in these scenarios. In addition, the fast manufacturing process is very well suited for adjustments that have to be made due to the rapid decrease in swelling after a fracture. Further advances in materials technology are also making it possible to use increasingly stiff, heat-resistant, and skin-compatible materials. The good functionality of such a material was shown in the present work. The fact that this material can be processed, not only at the specimen level, under perfect conditions using printers with industrial standards was further demonstrated. For future work, a stiffness comparison of the resulting structure with other leg splints from different works is planned to verify the anticipated better efficiency. Furthermore, a verification of the usability with the help of patient gait recordings will be considered in future research.

## Figures and Tables

**Table 1:** Overview of the identified material parameters for PETG-CF15.

$E_{x}$	$E_{y}$	$E_{z}$	$\nu_{xy}$	$\nu_{yz}$	$\nu_{xz}$	$G_{xy}$	$G_{yz}$	$G_{xz}$
MPa	MPa	MPa	-	-	-	MPa	MPa	MPa
8153	1949	1549	0.31	0.17	0.36	1096	642	1120

**Table 2:** Identified manufacturing parameters for PETG-CF15 material.

**Hotend Temperature**	**Bed Temperature**	**Layer Fan Speed**	**Perimeter Print Speed**	**Infill Print Speed**
260 °C	100 °C	60%	30 mm·s^−1^	25 mm·s^−1^
**First Layer Print Speed**	**General Print Speed**	**Support Type**	**Infill Density**	**Slicer Engine**
8 mm·s^−1^	20 mm·s^−1^	Organic	100%	Arachne
**Layer Thickness**	**Extrusion Flow Rate**	**Nozzle Diameter**	**Brim Thickness**	**Infill Pattern**
0.2 mm	92%	0.6 mm	3 mm	Concentric

## Data Availability

Not applicable.
